# Smoflipid Is Better Than Lipofundin for Long-Term Neurodevelopmental Outcomes in Preterm Infants

**DOI:** 10.3390/nu13082548

**Published:** 2021-07-26

**Authors:** I-Lun Chen, Chih-Hsing Hung, Hsin-Chun Huang

**Affiliations:** 1 Department of Pediatrics, Kaohsiung Chang Gung Memorial Hospital and Chang Gung University, College of Medicine, Kaohsiung 83301, Taiwan; memeo1013@hotmail.com; 2 School of Traditional Chinese Medicine, College of Medicine, Chang Gung University, Linkou 33302, Taiwan; pedhung@gmail.com

**Keywords:** docosahexaenoic acid, nutrition, fish oil, parenteral nutrition, lipid emulsion

## Abstract

Neurodevelopmental morbidities developed more commonly in low-birth-weight premature infants. We sought to determine the effects of different lipid emulsions on the neurodevelopmental outcomes of children born prematurely. This retrospective cross-sectional study had two intervention legs, Lipofundin^®^ MCT/LCT (LIPO) versus Smoflipid^®^ (SMOF), which are mainly differentiated by fish oil. Data of premature neonates born between 2001 and 2015 from the research database of Chang Gung Memorial Hospital with corresponding individual medical records up to July 2020 were analyzed. Long-term neurodevelopmental outcomes were defined by the international classification of disease codes −9 or −10. The prevalence of diseases was compared between LIPO and SMOF groups at five and five years old and further analyzed by stratification of 1500 g birth weight. The LIPO and SMOF groups each included 1120 neonates. Epilepsy, cerebral palsy, developmental disorder and attention-deficit hyperactivity disorder (ADHD) were significantly decreased at age two years in the SMOF group, and epilepsy, language delay (LD), ADHD and autism spectrum disorder (ASD) were significantly decreased in the SMOF group at age five years. In children with birth weight < 1500 g, ADHD was decreased in the SMOF group at ages two and five years, and ASD was decreased in the SMOF group at age five years. In children with birth weight ≥ 1500 g, epilepsy, LD and ADHD were decreased in the SMOF group at age two years. LD was decreased in the SMOF group at age five years. We conclude that lipid emulsions with fish oil improve the neurodevelopmental outcomes of children born prematurely.

## 1. Introduction

Data from the World Health Organization reveal that approximately 15 million children are born prematurely each year worldwide [1]. In the last two decades, advanced management of high-risk pregnancies and perinatal care has improved the survival of extremely low birth weight (ELBW, referring to birth weight less than 1000 g) and extremely preterm birth (less than 28 weeks gestational age) markedly in neonates born at 23- and 24-weeks’ gestation [2]. However, neurodevelopmental impairments are still known to increase with decreasing gestational age. Comparisons of data between 1997–2001 and 2002–2006 from the Premature Foundation of Taiwan showed decreased incidence of cerebral palsy (CP) in preterm babies born at 31 to 33 weeks but increased incidence from 3.8% to 12.9% (*p* = 0.0134) for premature infants with 501–750 g birth weight [3]. Till now, extremely preterm children are still shown to be at risk of severe neurodevelopmental impairment [4]. 

Aside from cognitive and motor deficits, behavioral and psychological problems are also more likely for children born preterm than those born at term [5]. Data of extremely preterm infants extracted from the National Institute of Child Health and Human Development showed that one-third to one-quarter had behavioral and socioemotional competence deficits at 18 to 22 months corrected age [5]. Studies revealed that nutrition intake in the early life of very low birth weight infants was essential not only for growth but also for neurodevelopment. Human brain development begins in the third trimester of pregnancy and extends to six years old when brain size reaches approximately 90% of adult volume. Cumulative protein intake in early nutrition had a positive relationship with brain size and white matter maturation at term equivalent age [6]. Aggressive protein and energy intake in the first week of life contributed to better language development than conventional nutrition [7]. In preterm neonates, higher blood docosahexaenoic acid (DHA) levels at birth and near-term were associated with larger cortical grey matter, deep grey matter and brainstem volumes, which were associated with improved language and motor scores at 30–36 months of corrected age [8].

Fish oil is rich in α-linolenic acid (omega-3), such as DHA and eicosapentaenoic acid (EPA). DHA can be transferred to the fetus through the placenta and cross the blood-brain barrier and is rapidly accumulated in the fetal brain to promote brain growth during the third trimester of pregnancy [9]. Humans are biologically limited when it comes to synthesizing DHA [10]; thus, maternal DHA intake is likely to highly influence DHA concentration in the fetus. DHA concentration in breast milk varies by consumption, especially depending on the intake of marine foods or DHA supplementation [11]. The DHA found in breast milk has a worldwide mean of 0.32 ± 0.22% [12]. Taiwan is an island country and the percentages of DHA and EPA in Taiwanese human milk are 0.79% and 0.17%, respectively, which are higher than those in western countries [13]. However, parenteral nutrition supplementation is commonly used for preterm neonates in their early life because of their immature gastrointestinal systems. Lipids used within the first two days of life of preterm babies are safe and well-tolerated, providing energy and essential fatty acids before full enteral feeding, which is necessary for central nervous system development [14]. In an animal study, parenterally administered lipid emulsions enriched with DHA and arachidonic acid resulted in increased cortical DHA and improved body composition without affecting short-term neurodevelopmental outcomes [15].

DHA is accumulated from the mother and then intake continues postnatally, derived from the diet [16]. Preterm birth interrupts maternal DHA absorption, which results in depriving preterm babies of DHA. Thus, it is essential to supply it through parenteral or enteral lipids. In Chang Gung Memorial Hospital (CGMH), Lipofundin^®^ MCT/LCT, mainly composed of soybean oil and medium-chain triglycerides, was used before 2010. After that year, Smoflipid^®^, made up of soybean oil, olive oil, fish oil and medium-chain triglycerides, has been used instead. The compositions of the main fatty acids in Lipofundin^®^ MCT/LCT and Smoflipid^®^ are shown in Table 1. This study aimed to compare the long-term neurodevelopmental outcomes of preterm neonates who received Lipofundin^®^ MCT/LCT or Smoflipid^®^ by retrospectively reviewing data from the Chang Gung research database.

## 2. Materials and Methods

### 2.1. Data Source

This multi-center cross-sectional hospital-based study was conducted using data from the Chang Gung research database, which provides comprehensive computerized information from all patients who have ever visited CGMH. CGMH has six branches, including two tertiary medical centers. The branches are located in the north, center and south of Taiwan, and occupy more than 10% of the medical costs of the National Health Insurance of Taiwan. All premature babies who were born between 2001 and 2015 and met the international classification of disease (ICD)-9 codes between 765.20 and 765.28, and that had received Lipofundin^®^ MCT/LCT or Smoflipid^®^, were enrolled in this study. These enrolled babies were born either at CGMH or born at other hospitals, but all were admitted to CGMH within three days after birth. Patients who died during their first admission or were denied follow-up visits after first discharge were excluded. Patients’ medical records were reviewed retrospectively until July 2020 for neurodevelopmental outcomes, including language delay (LD) (ICD-9 code: 3153, ICD-10 code: F80 and H93.25), developmental disorder (ICD-9 code: 3154, 3155, 3158, 3159, ICD-10 code: F82, F88, F89, F81.9), CP (ICD-9 code: 343.9, ICD-10 code: G80.0–G80.9), autism spectrum disorder (ASD; ICD-9: 299, ICD-10: F84.0) and attention-deficit hyperactivity disorder (ADHD; ICD-9: 314.01, ICD10: F90.1, F90.2, F90.9). Covariate diseases were documented as meningitis (ICD-9: 00321, 0130, 01300, 320–324, 047–049, 053, 0531, 05310, 05472, 0721, 09042, 11283, ICD-10: G03.9), perinatal asphyxia (ICD-9: 768, 768.2–768.6, 768.9, 7990), hydrocephalus (ICD-9: 331.3, 331.4, 742.3, ICD-10: G91), intraventricular hemorrhage (IVH; ICD-9: 7721, ICD-10: P52), periventricular leukomalacia (PVL; ICD-9: 779.7, ICD-10: P91.2), patent ductus arteriosus (PDA; ICD-9: 7470, ICD-10: Q25), retinopathy of prematurity (ROP; ICD-9: 36216, 36210, 36221, ICD-10: H35), bronchopulmonary dysplasia (BPD; ICD-9: 7707, ICD-10: P270, P271, P278) and necrotizing enterocolitis (NEC; ICD-9: 77750, 7775, 77751, 77752, 77753, ICD-10: P77, P771, P772, P773, P779). LD, ASD and ADHD were diagnosed by pediatric psychologists, whereas developmental disorders and CP were diagnosed by pediatric neurologists. Developmental disorders were diagnosed based on at least two delays of motor function, speech, language, cognitive, play or social skills. A speech-specific delay was defined as a language delay. PVL and IVH were documented by both ICD codes and neurosonographic reports. Surgical records were also reviewed if PDA ligation and laser surgery, scleral buckling or bevacizumab injection for ROP had been performed. All covariate diseases occurred before the diagnosis of neurodevelopmental outcomes. Demographic data were collected, including gestational age, birth weight, birth length, head circumference, body temperature at admission and APGAR scores at 1 and 5 min after birth.

### 2.2. Nutritional Strategies

The nutrition guidelines for preterm babies at CGMH follow the recommendations of the American Academy of Pediatrics. The American Academy of Pediatrics Committee on Nutrition advises that the nutritional goal of preterm infants is to achieve postnatal growth that approximates the growth of a normal fetus at the same postconceptional age [17]. The recent practice recommends starting protein intake between 24 and 48 h of life at 0.5 g/kg/day and then adding a lipid emulsion at 0.5 g/kg/day 24 h later; these would both be increased by 0.5 g/kg/day increments to 3.0 to 3.5 g/kg/day [18]. In the study period, the integration of total parenteral nutrition (TPN) in each of the six branches of CGMH was similar. The lipid emulsions used for preterm neonates in neonatal intensive care units (NICUs) were Lipofundin^®^ MCT/LCT (Braun Melsungen AG, Melsungen, Germany) or Smoflipid^®^ (SMOF; Fresenius Kabi USA, Melrose Park, IL, USA). Every neonate only received one of those; a mixed lipid emulsion was not administered. The feeding protocol in the NICU was to introduce breast milk soon after birth. For those mothers who were unable to express sufficient breast milk for full enteral feeding, or when donor human milk is not available, preterm formula was used instead. Usually, breast milk/preterm formula was started from trophic feeding 10mL/kg/day for 3–5 days and then advanced to a feeding amount of 10–20 mL/kg/day in ELBW infants or 20–30 mL/kg/day in very low birth weight (VLBW, referring to birth weights less than 1000 g) infants. Low-osmolality preterm formula, at a 20 kcal/oz concentration, was used for very low birth weight neonates and advanced to 24 kcal/oz later. When enteral feeding reached 100 mL/kg/day, the lipid emulsion supplementation would be discontinued and fortification with fortified human milk was initiated. The enteral ferrous-drop supplement was started at 4–8 weeks after birth to prevent anemia of prematurity. Micronutrients were not added into TPN until 2016 at CGMH.

### 2.3. Statistical Analysis

**Figure 1:**
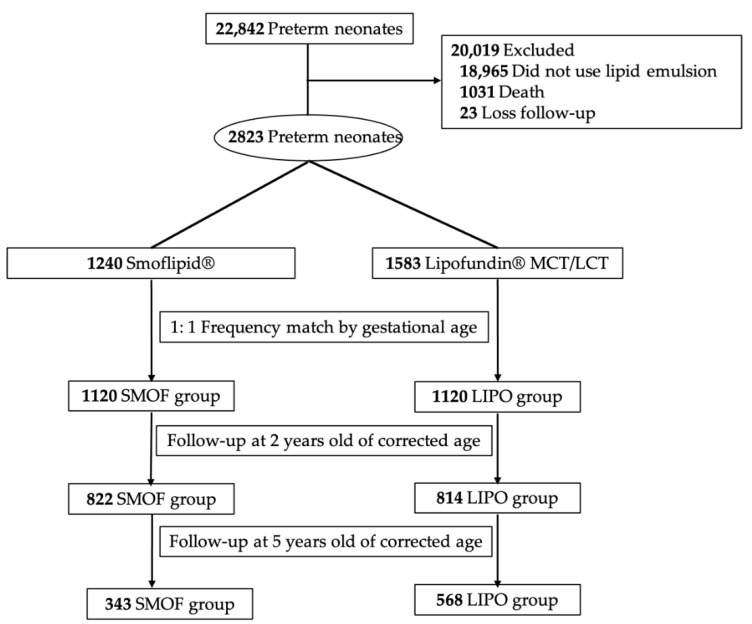
Flow chart of included and excluded patients.

All patients who received Smoflipid^®^ or Lipofundin^®^ MCT/LCT were divided into a SMOF and a LIPO group, respectively. The gestational age was entered into a logistic regression to create a propensity score for matching. These two groups were matched by a frequency of 1:1 on the basis of gestational age to control its effect on neurodevelopmental outcomes. A flow chart of included and excluded patients is presented in Figure 1.

Demographic and clinical (morbidity) data were compared between the SMOF and LIPO groups using the chi-square test for dichotomous variables and the *t*-test for continuous data. Neurodevelopmental outcomes are shown at two points: two and five years of corrected age. Patients in each group were further stratified by birth weight and divided into ≥1500 g and <1500 g subgroups. Neurodevelopmental outcomes were further analyzed by logistic regression after adjusting for comorbidities. All data were analyzed statistically using IBM SPSS statistics software (IBM Corp., Armonk, NY, USA) and *p* < 0.05 was considered statistically significant.

## 3. Results

From the year 2001 to 2015, there were 22,842 premature neonates admitted to CGMH. A total of 18,965 neonates who did not receive lipid emulsions were excluded. Of the remaining 3877 neonates, 1031 neonates died during the first admission and 23 were lost to follow-up. Among the surviving infants, 1240 and 1583 infants received Smoflipid^®^ or Lipofundin^®^ MCT/LCT for lipid supplementation after birth. After 1:1 frequency matching of gestational age, there were 1120 infants in each group. The demographic and clinical characteristics of these two groups are shown in Table 2. Body temperature at admission, APGAR score at 1 and 5 min after birth and the morbidities of PDA, ROP, BPD and NEC were significantly different between the two groups. The neurodevelopmental outcomes of the two groups are shown in Table 3 and Table 4. When premature neonates were two years old according to their corrected age, there remained 822 and 814 children in the SMOF and LIPO groups. The prevalence of epilepsy, CP, developmental delay and ADHD were significantly lower in the SMOF group than in the LIPO group, even after adjusting for significant comorbidities (body temperature, APGAR score at 1 and 5 min, PDA, ROP, BPD and NEC; *p* = 0.0168, 0.0179, 0.0335, 0.00001, respectively). These children were further stratified by their birth weights into ≥1500 g and <1500 g subgroups. In children with birth weights of less than 1500 g, ADHD in the SMOF group was less prevalent than that in the LIPO group (*p* = 0.0005, OR: 4.189). In children with birth weights of more than 1500 g, epilepsy, LD and ADHD were less prevalent in the SMOF group than that in the LIPO group (*p* = 0.0245, OR: 2.612; *p* = 0.0202, OR: 2.252; *p* = 0.0332, OR: 2.481). When the premature children were five years old, 343 and 568 children were left in the SMOF and LIPO groups. The prevalence of epilepsy, LD, ADHD and ASD were significantly lower in the SMOF group after adjusting for comorbidities (*p* = 0.0274, 0.0344, 0.0035, 0.0005, respectively). In children with birth weights of less than 1500 g, the prevalence of ADHD and ASD in the SMOF group was less than that in the LIPO group (*p* = 0.0172, OR: 2.912; *p* = 0.0164, OR: 12.793). In children with birth weights of more than 1500 g, the prevalence of LD in the SMOF group was less than that in the LIPO group (*p* = 0.0260, OR: 2.308).

## 4. Discussion

In this large cohort study, we found that administration of Smoflipid^®^ parenterally in the early life of premature neonates was associated with a difference in long-term neurodevelopment outcomes, including reducing epilepsy, CP, LD, developmental disorder, ADHD and ASD. Remarkably, in neonates with birth weights of less than 1500 g, developmental psychiatric disorders, ADHD and ASD were significantly decreased in the SMOF group compared to the LIPO group. A previous study also showed that Bayley-III language scores <85 and <70 were significantly lower in the SMOF group [19]. A pilot trial found a clinically significant improvement in ASD symptoms in children who received omega-3–6–9 supplementation [20].

Extremely premature babies cannot be enterally fed smoothly in their early life. Premature babies lose their standard placental supply of nutrients, have very low-fat stores and have immature digestive and metabolic functions [21], accounting for the EPA or DHA deficiency. DHA is one of the components of neural membranes and modulates their physicochemical properties and functions [22]. It can be found in synapses to influence neurotransmitters, gene expression and enzyme activity [23]. DHA also has anti-oxidative stress, anti-inflammatory and anti-apoptosis properties that diminish brain necrosis after hypoxic-ischemic injury by stabilizing intracellular ions between presynaptic and postsynaptic neurons [24]. Overall, the results of the above and our studies both demonstrate that DHA has a beneficial effect on neurodevelopment.

Preterm babies are born with life-threatening conditions that may cause brain injury, including respiratory distress, IVH, neonatal sepsis, PDA and NEC. It is worth mentioning that the prevalence of BPD was higher in the SMOF group than in the LIPO group in this study, which was in agreement with previous studies [25,26]. The survival rate was increasing in recent years so that a growing prevalence of BPD was observed synchronously. In addition, a multicenter randomized placebo-controlled clinical trial showed that DHA did not protect preterm infants from BPD [26]. Further studies will be needed to investigate the BPD issue. ELBW neonates have higher rates of brain damage and brain lesions, measured through decreased cortical gray matter volumes. A meta-analysis revealed that preterm-born children had an inferior cognitive ability when compared to those who were term-born, and had a higher risk of ADHD [27]. Functional MRI revealed that neural functional connectivity was diminished in fetuses that would later be born preterm versus term [28]. ADHD is a multifactorial disease, including inheritance and perinatal factors. Prematurity, low birth weight, born by cesarean section, pre-eclampsia, low APGAR score and maternal smoking during pregnancy are all shown to increase the risk of ADHD [29].

ASD is usually diagnosed around 2–3 years old when children present apparent signs. Impairments in social and communication skills and restricted and repetitive behaviors when it comes to interests and activities are two main characteristics of ASD. The prevalence of ASD in the very preterm population studied in Taiwan correlated to the 7% prevalence rate in a meta-analysis study of children born preterm [30,31]. The high prevalence of ASD in the preterm population might be caused by preterm birth and/or by alteration of immature brain growth, as genetic factors may play a role in both preterm birth and ASD [31]. The prevalence of ASD in school-aged children has been reported as significantly increasing from 2007 to 2011–2012 [32]. In Taiwan, the National Health Insurance provides free physical and growth assessments to all children during their visits for vaccinations. Suspected cases will be referred to pediatric psychologists for further evaluation. In this study, the prevalence of ASD below five years old was 6% (2001–2010), which correlated with other studies [30,31]. Intriguingly, the prevalence of ASD in premature infants after 2010 was decreasing to 0.6%. Though the perinatal care of premature neonates was more advanced after 2010 than before, the prevalence of IVH, PVL, hydrocephalus and meningitis were not found to be significantly changed (Table 2). As many morbidities may influence the outcomes, the neuropsychiatric outcomes in the SMOF group were still better than in the LIPO group after adjustment of multiple variables by logistic regression analysis. Thus, this study presented evidence of the benefit of fish oil for the developing brain in the premature population.

The strengths of the present study are the large cohort and long-term follow-up. Data were analyzed after matching gestational age and adjusting for morbidities of premature infants. Gestational age and small birth weight have a strong association with neurodevelopmental outcomes. Any management during admission could improve or worsen neurodevelopmental outcomes or other morbidities. Thus, if the sample size is small, or we do not adjust for morbidities before analysis, the results could be over-or underestimated. As the enrolled patients were from six branches of CGMH, the content of TPN for all premature neonates was similar. Moreover, the average durations of lipid therapy in the two groups were 17.1 and 15.2 days, respectively, without significant difference (Table 2). Besides, genetic factors, prenatal insults and the parents’ education levels and socioeconomic statuses were not analyzed in this study. Neonatal and maternal medical records cannot be linked, so the records of prenatal insults that induced premature delivery, and parents’ information were not available in this database. Genetic factors and parents’ socioeconomic statuses are known to contribute significantly to neurodevelopmental disorders, including LD, autism and ADHD [33], but they were not discussed in this study. Since the six branches of CGMH are distributed throughout northern, central and southern Taiwan, from rural to urban, their overall treatment populations may be representative of the general population. Furthermore, some patients were lost to follow-up at two and five years old. The possible reasons for this are the policy of Taiwan National Health Insurance and the vaccination schedule. In recent years, the government has encouraged medical centers to refer patients with mild morbidities to local hospitals or clinics. Patients who need to visit specialists (pediatric psychologists and neurologists) are still regularly followed up at our hospitals. Although we have excluded the infants who died during their first admission, when it comes to those who did not visit Chang Gung hospitals during their mild illness or those who died after their first admission, their data would not be collected in this database.

## 5. Conclusions

The results of this large cohort study have demonstrated that lipid emulsions with fish oil improve the long-term neurodevelopmental outcomes of children born prematurely. In particular, the prevalence of ADHD and autism were significantly decreased in neonates with birth weights below 1500 g. Though not comprehensively analyzed, big data analysis has been used reliably. Further investigation of more methodological rigor using a randomized controlled trial is needed.

## Figures and Tables

**Table 1:** Composition of main fatty acids in Smoflipid^®^ and Lipofundin^®^ MCT/LCT.

Fatty Acids	SMOFlipid^®^, 0.2 g/mL (%)	Lipofundin^®^ MCT/LCT, 0.2 g/mL (%)
Oleic acid	23 to 35	-
Linoleic acid	14 to 25	24 to 29
α-linolenic acid	1.5 to 3.5	2.5 to 5.5
Eicosapentaenoic acid	1 to 3.5	-
Docosahexaenoic acid	1 to 3.5	-
Medium-chain triglycerides	30	50

**Table 2:** The demographic and clinical characteristics of the SMOF and LIPO groups (*n* = 2240).

	SMOF Group (*n* = 1120)	LIPO Group (*n* = 1120)	*p* Value
Gestational age, mean (SD), week	32.15 (2.38)	32.15 (2.38)	1
Gender, no. (%)			
Female	521 (46.5)	503 (44.9)	0.42
Male	599 (53.4)	617 (55.1)	
Birth length, mean (SD), cm	41.4 (4.07)	40.8 (4.26)	0.0006
Birth weight, mean (SD), g	1759.8 (514.6)	1764.9 (558.7)	0.82
Body temperature, mean (SD), ℃	36.5 (0.58)	36.4 90.67)	0.0040
Head circumference, mean (SD), cm	29.6 (2.49)	29.5 (2.73)	0.47
AS1, mean (SD)	7.2 (1.74)	6.5 (2.01)	<0.0001
AS5, mean (SD)	8.7 (1.28)	8.2 (1.52)	<0.0001
Duration of lipid, mean (SD), day	17.1 (21.18)	15.2 (15.58)	0.05
Comorbidities, no. (%)			
Meningitis	2 (0.17)	6 (0.53)	0.29
Perinatal asphyxia	46 (4.10)	44 (3.93)	0.83
Hydrocephalus	16 (1.42)	23 (2.05)	0.33
Intraventricular hemorrhage	108 (9.64)	97 (8.66)	0.38
Periventricular leukomalacia	11(0.98)	17 (1.51)	0.34
Patent ductus arteriosus	88 (7.85)	186 (16.60)	<0.0001
PDA ligation	31 (2.76)	54 (4.82)	0.0149
Retinopathy of prematurity	308 (27.50)	394 (35.17)	0.0003
Surgical management for ROP	16 (1.42)	13 (1.16)	0.58
Bronchopulmonary dysplasia	133 (11.87)	82 (7.32)	0.0002
Necrotizing enterocolitis	18 (1.60)	7 (0.62)	0.0269

Abbreviations: AS1, Apgar score at 1 min; AS5, Apgar score at 5 min; PDA, patent ductus arteriosus; ROP, retinopathy of prematurity; SD, standard deviation.

**Table 3:** The neurodevelopmental outcomes of SMOF and LIPO groups at two years old as corrected age (*n* = 1636).

No (%)	SMOF Group (*n* = 822)	LIPO Group (*n* = 814)	Logistic Regression *		
			OR	95% CI	*p* Value
Epilepsy	26 (3.2)	45 (5.5)	2.111	1.144–3.895	0.0168
Cerebral palsy	15 (1.8)	33 (4.0)	2.444	1.166–5.121	0.0179
Language delay	51 (6.2)	67 (8.2)	1.291	0.835–1.995	0.25
Developmental delay	2016 (12.7)	153 (18.8)	1.408	1.027–1.931	0.0335
ADHD	20 (2.4)	68 (8.3)	3.063	1.732–5.416	0.0001
Autism	2 (0.2)	2 (0.2)	0.615	0.053–7.153	0.70
Birth weight < 1500 g	302 (36.7)	295 (36.2)			
Epilepsy	11 (3.6)	20 (6.8)	1.629	0.638–4.158	0.31
Cerebral palsy	6 (2.0)	18 (6.1)	2.681	0.885–8.121	0.0812
Language delay	35 (11.6)	33 (11.2)	0.928	0.505–1.705	0.81
Developmental delay	65 (21.5)	90 (30.5)	1.427	0.918–2.218	0.11
ADHD	11 (3.6)	37 (12.5)	4.189	1.874–9.365	0.0005
Autism	0 (0)	1 (0.3)			0.99
Birth weight ≥ 1500 g	520 (63.3)	519 (63.8)			
Epilepsy	15 (2.9)	25 (4.8)	2.612	1.131–6.030	0.0245
Cerebral palsy	9 (1.7)	14 (2.7)	2.061	0.758–5.604	0.16
Language delay	16 (3.0)	33 (6.4)	2.252	1.135–4.467	0.0202
Developmental delay	40 (7.7)	62 (12.0)	1.493	0.926–2.405	0.10
ADHD	9 (1.7)	31 (6.0)	2.481	1.075–5.724	0.0332
Autism	2 (0.4)	1 (0.2)	0.000	-	0.99

Abbreviations: ADHD, attention deficit and hyperactivity disorder; CI, confidence interval; OR, odds ratio. * Adjusted for Apgar score 1 and 5, body temperature, bronchopulmonary dysplasia, patent ductus arteriosus, retinopathy of prematurity, necrotizing enterocolitis.

**Table 4:** The neurodevelopmental outcomes of SMOF and LIPO groups at five years old as corrected age (*n* = 911).

No. (%)	SMOF Group (*n* = 343)	LIPO Group (*n* = 568)	Logistic Regression *		
			OR	95% CI	*p* Value
Epilepsy	12 (3.5)	38 (6.7)	2.635	1.114–6.232	0.0274
Cerebral palsy	14 (4.0)	40 (7.0)	1.562	0.727–3.358	0.25
Language delay	31 (9.0)	87 (15.3)	1.706	1.040–2.799	0.0344
Developmental delay	65 (18.9)	134 (23.6)	1.101	0.746–1.625	0.63
ADHD	17 (5.0)	77 (13.5)	2.473	1.347–4.543	0.0035
Autism	2 (0.6)	35 (6.1)	7.055	1.581–31.486	0.0105
Birth weight < 1500 g	113 (32.9)	203 (35.7)			
Epilepsy	6 (5.3)	18 (8.8)	1.765	0.584–5.335	0.31
Cerebral palsy	5 (4.4)	26 (12.8)	1.606	0.503–5.126	0.42
Language delay	17 (15.0)	40 (19.7)	1.360	0.674–2.741	0.39
Developmental delay	35 (30.9)	69 (34.0)	0.948	0.535–1.680	0.86
ADHD	9 (7.9)	36 (17.7)	2.912	1.209–7.012	0.0172
Autism	1 (0.8)	21 (3.7)	12.793	1.595–102.581	0.0164
Birth weight ≥ 1500 g	230 (67.0)	365 (64.3)			
Epilepsy	6 (2.6)	20 (5.5)	4.481	0.963–20.847	0.06
Cerebral palsy	9 (3.9)	18 (4.9)	1.412	0.504–3.959	0.51
Language delay	14 (6.1)	46 (12.6)	2.308	1.105–4.820	0.0260
Developmental delay	30 (13.0)	64 (17.5)	1.306	0.751–2.272	0.34
ADHD	8 (3.4)	41 (11.2)	2.268	0.952–5.403	0.06
Autism	1 (0.4)	14 (3.9)	2.351	0.244–22.679	0.46

Abbreviations: ADHD, attention deficit and hyperactivity disorder; CI, confidence interval; OR, odds ratio. * Adjusted for Apgar score 1 and 5, body temperature, bronchopulmonary dysplasia, patent ductus arteriosus, retinopathy of prematurity, necrotizing enterocolitis.

## Data Availability

The data presented in this study are available on request from the corresponding author. The availability of the data is restricted to investigators based in academic institutions.

## Abbreviations

ADHD—Attention Deficit and Hyperactivity Disorder; ASD—Autism Spectrum Disorder; BPD—Bronchopulmonary Dysplasia; CGMH—Chang Gung Memorial Hospital; CP—Cerebral Palsy; DHA—Docosahexaenoic Acid; ELBW—Extremely Low Birth weight; EPA—Eicosapentaenoic Acid; ICD—International Classification of Disease; IVH—Intraventricular Hemorrhage; LD—Language Delay; NEC—Necrotizing Enterocolitis; NICHD—National Institute of Child Health and Human Development; PDA—Patent Ductus Arteriosus; PVL—Periventricular Leukomalacia; ROP—Retinopathy Of Prematurity; TPN—Total Parenteral Nutrition; VLBW—Very Low Birth weight.
